# Adoption of C-reactive protein rapid tests for the management of acute childhood infections in hospitals in the Netherlands and England: a comparative health systems analysis

**DOI:** 10.1186/s12913-024-10698-6

**Published:** 2024-03-19

**Authors:** Juan Emmanuel Dewez, Ruud G. Nijman, Elizabeth J. A. Fitchett, Edmond C. Li, Queena F. Luu, Rebecca Lynch, Marieke Emonts, Ronald de Groot, Michiel van der Flier, Ria Philipsen, Stefanie Ettelt, Shunmay Yeung

**Affiliations:** 1 Clinical Research Department, London School of Hygiene & Tropical Medicine, London, UK; 2 Section of Paediatric Infectious Diseases, Department of Infectious Diseases, Imperial College London, London, UK; 3 Institute of Global Health Innovation, Department of Surgery and Cancer, Faculty of Medicine, Imperial College, London, UK; 4 Wellcome Centre for Cultures and Environments of Health, University of Exeter, Exeter, UK; 5 Paediatric Immunology, Infectious Diseases & Allergy, Great North Children’s Hospital, Newcastle Upon Tyne Hospitals NHS Foundation Trust, Newcastle Upon Tyne, UK; 6 Translational and Clinical Research Institute, Newcastle University, Newcastle Upon Tyne, UK; 7 Section of Paediatric Infectious Diseases, Laboratory of Medical Immunology, Radboud Centre for Infectious Diseases, Radboud Institute for Molecular Life Sciences, Radboud UMC, Nijmegen, the Netherlands; 8 Paediatric Infectious Diseases and Immunology, Amalia Children’s Hospital, Radboud UMC, Nijmegen, the Netherlands; 9 Paediatric Infectious Diseases and Immunology, Wilhelmina Children’s Hospital, University Medical Center Utrecht, Utrecht, the Netherlands; 10 Department of Health Services Research and Policy, London School of Hygiene & Tropical Medicine, London, UK; 11 Prognos AG, Basel, Switzerland; 12 Department of Paediatrics, St Mary’s Imperial College Hospital NHS Trust, London, UK

**Keywords:** Comparative health systems analysis, NASSS framework, C-reactive protein, Point-of-care tests, The Netherlands, England, Acute childhood infections, Hospital care

## Abstract

**Background:**

The adoption of C-reactive protein point-of-care tests (CRP POCTs) in hospitals varies across Europe. We aimed to understand the factors that contribute to different levels of adoption of CRP POCTs for the management of acute childhood infections in two countries.

**Methods:**

Comparative qualitative analysis of the implementation of CRP POCTs in the Netherlands and England. The study was informed by the non-adoption, abandonment, spread, scale-up, and sustainability (NASSS) framework. Data were collected through document analysis and qualitative interviews with stakeholders. Documents were identified by a scoping literature review, search of websites, and through the stakeholders. Stakeholders were sampled purposively initially, and then by snowballing. Data were analysed thematically.

**Results:**

Forty-one documents resulted from the search and 46 interviews were conducted. Most hospital healthcare workers in the Netherlands were familiar with CRP POCTs as the tests were widely used and trusted in primary care. Moreover, although diagnostics were funded through similar Diagnosis Related Group reimbursement mechanisms in both countries, the actual funding for each hospital was more constrained in England. Compared to primary care, laboratory-based CRP tests were usually available in hospitals and their use was encouraged in both countries because they were cheaper. However, CRP POCTs were perceived as useful in some hospitals of the two countries in which the laboratory could not provide CRP measures 24/7 or within a short timeframe, and/or in emergency departments where expediting patient care was important.

**Conclusions:**

CRP POCTs are more available in hospitals in the Netherlands because of the greater familiarity of Dutch healthcare workers with the tests which are widely used in primary care in their country and because there are more funding constraints in England. However, most hospitals in the Netherlands and England have not adopted CRP POCTs because the alternative CRP measurements from the hospital laboratory are available in a few hours and at a lower cost.

**Supplementary Information:**

The online version contains supplementary material available at 10.1186/s12913-024-10698-6.

## Background

Fever is a common reason for children to present to hospitals [1, 2]. Most febrile children have self-limiting infections but differentiating the few febrile children with severe bacterial infections from those with self-limiting illness is difficult because the clinical features of infections in children are often non-specific [3]. Consequently, febrile children may be prescribed unnecessary antibiotics, subjected to invasive tests, and admitted for monitoring whilst awaiting microbiology results [4]. This causes pain, distress, and inconvenience, and may contribute to antimicrobial resistance (AMR) [5].

Point-of-care tests (POCTs) have been widely advocated to reduce the use of antibiotics [6]. This is because they can be performed easily in the consultation room and provide rapid results. Using POCTs may also reduce hospital admissions and optimise the use of resources in general [7].

There are a number of POCTs that can be used in the clinical management of acute infections in children, although their impact varies [8]. These include urine dipsticks to diagnose urinary tract infections, rapid throat swabs to identify Group A Streptococcal infections, and C-reactive protein (CRP) POCTs performed on blood from a finger prick to differentiate bacterial from viral infections [9]. CRP is one of the most used biomarkers in the management of febrile children, but there are substantial ongoing efforts to develop new blood tests to determine the cause of fever with more precision [10, 11].

The COVID-19 pandemic has contributed to an increased awareness about the role and importance of diagnostic tests, particularly POCTs. This was not only among healthcare professionals but also among members of the public, who learned to use and interpret the results of COVID-19 POCTs. This could lead to the perception that the use of POCTs will increase in clinical practice. However, the adoption of POCTs can be complex and be influenced by multiple factors, such as the engagement of early adopters and the role of clinical guidelines in determining re-imbursement schemes, which played an important role in the adoption of CRP POCTs in primary care in the Netherlands [12].

The availability and use of CRP POCTs in hospitals varies across Europe [13, 14]. To inform the effective implementation of current and future POCTs in hospitals, understanding the reasons for this variation is important but is currently lacking.

The aim of this study was to generate an in-depth understanding of the factors that contributed to different levels of adoption of CRP POCTs for hospital-level management of acute childhood infections in two European countries.

## Methods

**Fig. 1:**
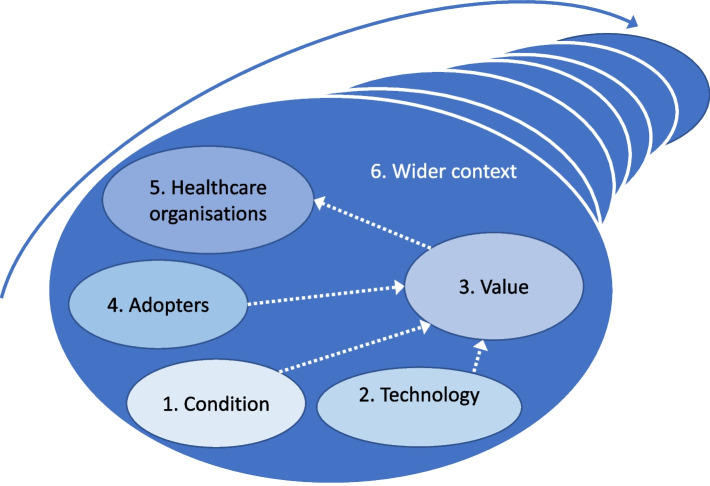
The non-adoption, abandonment, spread, scale-up and sustainability of healthcare technologies (NASSS) framework (adapted from Greenhalgh et al.) [15]

A comparative qualitative analysis based on two country case studies of the implementation of CRP POCTs was conducted. Qualitative methods were used because they are best suited to study phenomena such as the introduction of diagnostics in hospitals which is multifaceted and involves multiple actors and processes in a wider national context. The design of the study was informed by the non-adoption, abandonment, spread, scale-up, and sustainability (NASSS) of healthcare technologies framework [15]. The NASSS framework was developed to identify factors that contribute to the adoption of innovations in healthcare services by assessing the complexity of seven domains: (1) the condition or illness; (2) the technology; (3) the value of the innovation for developers and users; (4) the adopters and whether the innovation implied a change in their identity and practices; (5) the healthcare organisations where the innovation is implemented, their readiness for this innovation, how the innovation changes the organisations’ routines, and the work needed to adopt, fund, and normalise the innovation; (6) the wider context including the policy and regulatory contexts, the role of professional bodies and interorganisational networking; and (7) the adaptation over time of the innovation, its use, and the organisations (Fig. 1).

The countries were selected to allow a “most similar" type of comparison [16], i.e., the countries were different for the outcome of interest (the availability of CRP POCTs in hospitals) but were similar in other aspects such as the care pathways for acute fever in children, the role of hospitals in this care pathways, the source of hospital funding, and the share of the country wealth that is invested in healthcare. A benefit of a “most similar” approach is that it makes it easier to control for factors that are similar in the two countries (and thus do not contribute to the different outcome of interest) and to focus only on factors that are different and may contribute to the outcome. The selected countries were the Netherlands and England because in a previous cross-sectional survey we estimated that the availability of CRP POCTs in hospitals was different, the tests being available in 18% of hospitals in the Netherlands versus 5% in England [14]. Moreover, the two countries are similar in other important factors such as general practitioners (GPs) being the recommended first point of care before hospitals in both countries, most (~ 80%) of health expenditure being funded by public sector sources in both countries (mainly from compulsory social health insurance in the Netherlands and from general taxation in England) [17], and both countries investing approximately 10% of gross domestic product on healthcare [18]. An additional reason for choosing these countries was that we previously conducted a similar qualitative study comparing the adoption of CRP POCTS in the same countries but at primary care level [12], and conducting a study in hospital settings would complement the findings of the primary care study and provide a comprehensive understanding of why the tests are more commonly available in the Netherlands at primary care and hospital levels compared to England.

Data were obtained through two approaches: 1) a review of publicly available documents and 2) qualitative semi-structured interviews with stakeholders. The document analysis sought to initially explore the wider health systems of the countries and to inform the identification of relevant stakeholders and the development of topic guides (Supplementary Material). This was followed by interviews with stakeholders and then additional analyses of documents suggested during the interviews of stakeholders. The iterative combination of these two methods allowed triangulation of data for two purposes: 1) to cross-validate findings and 2) to extend the understanding of findings.

The criteria for documents to be included in the documents review were that they had to pertain to the adoption of CRP POCTs in one or the two countries and had to be published after 2000. Documents in English and Dutch were included. Documents included peer-reviewed publications in medical journals, clinical guidelines, reports from healthcare organisations, health systems reviews, and policies. Documents were identified through a three-pronged approach. A scoping review of the literature was conducted by JED by searching Pubmed and Google on the following topics: epidemiology of febrile children; the clinical performance, clinical effectiveness, and cost effectiveness of CRP POCTs; the adoption of the tests in the two countries; and the main characteristics of the countries’ health systems. This was followed by an extensive search of relevant healthcare organisations’ websites (including clinical commissioning groups; professional associations of clinicians and industry; clinical guidelines development bodies; local, national, and European health authorities; independent bodies advising these authorities; independent bodies assessing healthcare interventions; health insurance companies; and the in vitro diagnostics industry). Finally, documents were also obtained through interviewees’ recommendations and through attendance to relevant seminars and conferences.

The criteria for stakeholders to be invited to participate to the qualitative interviews were that they had to be experts of at least one domain of the NASSS framework pertaining to the adoption of CRP POCTs in hospitals in one of the two countries. We also ensured that we had at least one representative of the three level of health systems: micro (stakeholders who used/could use CRP POCTs), meso (stakeholders directly involved in the implementation of diagnostics in hospitals) and macro (stakeholders involved in the wider national context).

Initial interviewees were sampled purposively. This was followed by snowball sampling to identify additional stakeholders that could provide insights on domains of the NASSS framework not covered in the initial interviews. In the Netherlands, the initial interviewees were based in Nijmegen because members of the research team (RD, MVF, RP) were based there. Further stakeholders were based in Eindhoven and Leusden. RD, MVF, RP identified potential participants, based on the inclusion criteria, among members of staff of their hospitals and experts of the topic of interest who they knew from previous collaborations. Potential participants were contacted by email or telephone to ascertain their interest in being interviewed. Those who agreed were followed-up by JED who provided a participant information sheet, obtained written informed consent, and arranged the interview date. In England, interviewees worked in Newcastle and London. Paediatricians and nurses were interviewed as part of a related project led by JED and SY aiming to explore the views of clinicians about using POCTs in general (not only CRP POCTs) in children [19]. The other stakeholders were identified through searching authors of medical articles on the use of CRP POCTs in England, by attending conferences about the adoption of diagnostics in the National Health Service (NHS) and by snowballing. JED conducted all the interviews in the Netherlands and the interviews in England with stakeholders other than paediatricians and nurses. Paediatricians and nurses in England were interviewed by EL and QL. SY participated in two interviews and RGN participated in one interview in the Netherlands. The interviewers did not know participants beforehand. Face-to-face audio recorded interviews took place at the respondents’ workplace between June 2018 and February 2020, and by videoconference between March 2020 and January 2022 because of restrictions due to the COVID-19 pandemic. Only the interviewers and the participants were present during the interview. All interview records were transcribed verbatim by a research assistant, EL, QL, or JED. Field notes were taken after each interview. One transcript was returned to a participant who requested this; no corrections were made. One participant was recontacted to clarify the information provided in the interviews. No repeat interviews were conducted.

The data from documents and from interview transcripts were analysed thematically. The analysis was deductive based on the seven domains of the NASSS framework. JED extracted data from the documents and from interview transcripts and collated them per NASSS domain using matrices in Excel, including alternative views when available. Data from the two countries were collated separately. EJAF independently assessed whether each extract was assigned to the most relevant NASSS domains. Discrepancies were resolved through discussion and consensus between JED and EJAF. A summary of each domain was then produced by JED. JED compared side by side the summaries from the two countries for each domain to identify similarities and discrepancies that could contribute to the difference in the outcome of interest and produced a comparative summary per domain. The comparative summaries were circulated to all members of the research team to check whether they were clear, coherent, internally consistent, and credible within the context of hospital paediatric care in the two countries. The latter was possible thanks to the combined expertise of the research team about paediatric care in the Netherlands and England. There were minor suggestions by the research team to improve the clarity of the text and minor comments on inconsistencies across summaries that were clarified through discussions. JED amended the comparative summaries and recirculated them. All research team members agreed on the final version. Data saturation was considered reached when all domains of the NASSS framework were covered and each domain was clearly understood. Participants did not provide feedback on the findings.

## Results

Forty-one documents including research publications, clinical guidelines, proceedings of workshops, health services assessments, health systems reviews, and policies were included in the analysis (Table 1). A total of 46 stakeholders were interviewed. This included healthcare workers (nurses, paediatricians, and laboratory staff) from four hospitals (two hospitals in each country). CRP POCTs had been used in the emergency department (ED) of one of the hospitals in England as part of a pilot study. One hospital in the Netherlands was about to implement CRP POCTs in its ED and in the two remaining hospitals the tests were never used, nor were there plans to do so. Other stakeholders included representatives of a clinical commissioning group, a health insurance company, an interorganisational networking public body, and the in vitro diagnostics industry (Table 2). Four successive industry representatives did not reply to the invitation in England. Interviews lasted 31–75 min.

**Table 1:** Documents included in the analysis

Author and country	Title	Type of document	NASSS domains
van Ierland, 2011 [1]	Self-referral and serious illness in children with fever	Observational study aiming to compare febrile children referred by a general practitioner with those self-referred in the Netherlands	Domain 1
Sands, 2011 [2]	Medical problems presenting to paediatric emergency departments: 10 years on	Observational study aiming to describe the common medical presenting problems of children attending a paediatric emergency department	Domain 1
Nijman, 2013 [20]	Clinical prediction model to aid emergency doctors managing febrile children at risk of serious bacterial infections: diagnostic study	Diagnostic test accuracy study of a predictive model for the assessment of the risks of serious bacterial infections in children with fever at the emergency department in the Netherlands and England	Domain 1 and 7
Le Doare, 2014 [21]	Very low rates of culture-confirmed invasive bacterial infections in a prospective 3-year population-based surveillance in Southwest London	Observational study aiming to estimate the incidence, clinical characteristics, and risk factors for culture-confirmed invasive bacterial infections in England	Domain 1
O’Brien, 2019 [22]	CRP POCT to guide antibiotic prescribing in primary care settings for acute respiratory tract infections	Health technology assessment of CRP POCT	Domain 2
Van den Bruel, 2011 [23]	Diagnostic value of laboratory tests in identifying serious infections in febrile children	Systematic review of the diagnostic test accuracy of various biomarkers including CRP to predict serious bacterial infections	Domain 2
NVK, 2013 [24]	Bacterial meningitis	Guidelines from the Dutch College of Paediatrics on meningitis	Domain 2
NVK, 2013 [25]	Fever in secondary care in children aged 0–16 years	Guidelines from the Dutch College of Paediatrics on fever	Domain 2
NVK, 2021 [26]	Sepsis in children	Guidelines from the Dutch College of Paediatrics on sepsis in children	Domain 2
NVK, 2017 [27]	Prevention and treatment of early-onset neonatal infections	Guidelines from the Dutch College of Paediatrics on sepsis in neonates	Domain 2
NICE, 2010 [28]	Meningitis (bacterial) and meningococcal septicaemia in under 16 s: recognition, diagnosis, and management	Guidelines from the National Institute for Health and Care Excellence on meningitis in children	Domain 2
NICE, 2019 [29]	Fever in under 5 s: assessment and initial management	Guidelines from the National Institute for Health and Care Excellence on fever in children < 5 years	Domain 2
NICE, 2021 [30]	Neonatal infection: antibiotics for prevention and treatment	Guidelines from the National Institute for Health and Care Excellence on neonatal infections	Domain 2
NICE, 2007 [31]	Urinary tract infection in under 16 s: diagnosis and management	Guidelines from the National Institute for Health and Care Excellence on urinary infections in children	Domain 2
RCPCH, 2021 [32]	COVID-19—guidance for management of children admitted to hospital and for treatment of non-hospitalised children at risk of severe disease	Guidelines from the British Royal College of Paediatrics and Child Health on COVID-19	Domain 2
Oxford AHSN, 2017 [33]	Unique point of care blood test speeds up clinical decision-making, improves quality of care and reduces costs	Report of a pilot study to assess the effectiveness of CRP POCT use in children to reduce length of stay in EDs and costs of care in three hospitals	Domain 3
Cylus, 2015 [34]	United Kingdom health system review	In-depth review of the British health system	Domain 5 and 6
Kroneman, 2016 [35]	The Netherlands health system review	In-depth review of the Dutch health system	Domain 5 and 6
Maguire, 2011 [36]	Which urgent care services do febrile children use and why?	Observational study aiming to explore how parents navigate urgent and emergency care services when their child < 5 years old has a feverish illness	Domain 5
Mossialos, 2017 [37]	International profiles of healthcare systems, 2016	In-depth review of the Dutch health system	Domain 5
Luppa, 2018 [38]	Point-of-Care Testing Principles and Clinical Applications	Multi-country evaluation of POC testing in hospitals	Domain 5
BIVDA, 2016 [39]	Point of care testing environment survey report	Survey by a British in vitro diagnostics professional association about the readiness of NHS trusts to implement POCTs	Domain 5
van Stijn, 2012 [40]	Data Quality of the Dutch DBC Information System	MSc thesis assessing the data quality of the DBC information system within Dutch hospitals	Domain 5
Busse, 2011 [41]	Diagnosis related groups in Europe: moving towards transparency, efficiency, and quality in hospitals?	Review of European health financing schemes	Domain 5
Academy of Medical Sciences, 2021 [42]	Building a sustainable UK diagnostics sector	Summary report of a FORUM workshop	Domain 5
Dutch government, 2015 [43]	Tackling antimicrobial resistance, the Dutch one health approach	Summary of the Dutch antibiotic resistance policy	Domain 6
UK government, 2013 [44]	UK 5-year antimicrobial resistance strategy 2013 to 2018	British antimicrobial resistance plan	Domain 6
Monitor, 2014 [45]	Exploring international acute care models	Multi-country analysis of acute service line models by Monitor, the regulator for health services in England	Domain 6
NHS England, 2019 [46]	Clinically led review of NHS access standards	Report from the NHS National Medical Director on NH standards	Domain 6
Parkin, 2020 [47]	NHS maximum waiting time standards	Briefing from the House of Commons on the NHS waiting time standards	Domain 6
Carter, 2006 [48]	Report of the Review of NHS Pathology Services in England	Independent review of NHS pathology services	Domain 6
Royal College of pathologists, 2017 [49]	Consolidation of pathology services: lessons learnt	Report of accounts from members of the Royal College of Pathologists about their experience of consolidation of pathology services	Domain 6
Satta, 2018 [50]	Consolidation of pathology services in England: have savings been achieved?	Descriptive comparison of savings among consolidated and non-consolidated pathology services	Domain 6
Jeurissen, 2021 [51]	The market reform in Dutch health care	In-depth review of the healthcare market reforms in the Netherlands	Domain 6
Anderson, 2021 [52]	Re-laying the foundations for an equitable and efficient health and care service after COVID-19	LSE-Lancet Commission on the future of the NHS	Domain 6
OECD, 2020 [53]	Health spending*,* 2020	Report on health spending in countries member of the Organisation for Economic Cooperation and Development	Domain 6
European Commission, 2021 [54]	CE marking	Information for the public about the European Union’s single market standards	Domain 6
ROS Robuust [55]	Robuust for healthy collaboration	Information for the public about regional support to healthcare services collaboration in the Netherlands	Domain 6
AHSN, 2021 [56]	Academic Health Science Network: transforming lives through healthcare innovation	Information for the public about regional support to dissemination of healthcare innovation in England	Domain 6
de Vos-Kerkhof, 20153 [57]	Impact of a clinical decision model for febrile children at risk for serious bacterial infections at the emergency department	Clinical trial aiming to assess the impact of a clinical decision model for febrile children attending the emergency department in the Netherlands	Domain 7
van de Maat, 2020 [58]	Evaluation of a clinical decision rule to guide antibiotic prescription in children with suspected lower respiratory tract infection in The Netherlands	Clinical trial aiming to assess the impact of a clinical decision model for children with lower respiratory infections attending the emergency department in the Netherlands	Domain 7

**Table 2:** Characteristics of stakeholders

**Stakeholders**	**Netherlands**				**England**		**Main health system level**
	Non-hospital stakeholder	Hospital 1 (secondary hospital)	Hospital 2 (tertiary hospital)	Non-hospital stakeholder	Hospital 3 (tertiary hospital)	Hospital 4 (tertiary hospital)	
In vitro diagnostics industry representative	1			1			Macro
Health insurance company representative	1						Macro
Clinical commissioning group member				1			Macro
Reimbursement of healthcare expert	1						Macro
Health services interorganisational networking expert (AHSN)				1			Macro
Head of laboratory department		1	2				Meso
POCT manager						1	Meso
Head of emergency department		1			1		Meso
Emergency department nurse		1	2		1	2	Micro
Emergency department doctor			1		3	2	Micro
Paediatric infectious diseases doctor			1				Micro
General paediatrician		1	1		2	1	Micro
Paediatric trainee		1	1		6	6	Micro
ED trainee		1					Micro
Total		17			28		

*AHSN* Academic Health Science Network

The analysis identified similarities and differences in the seven NASSS domains between the two countries (Table 3) and are presented narratively below.

**Table 3:**
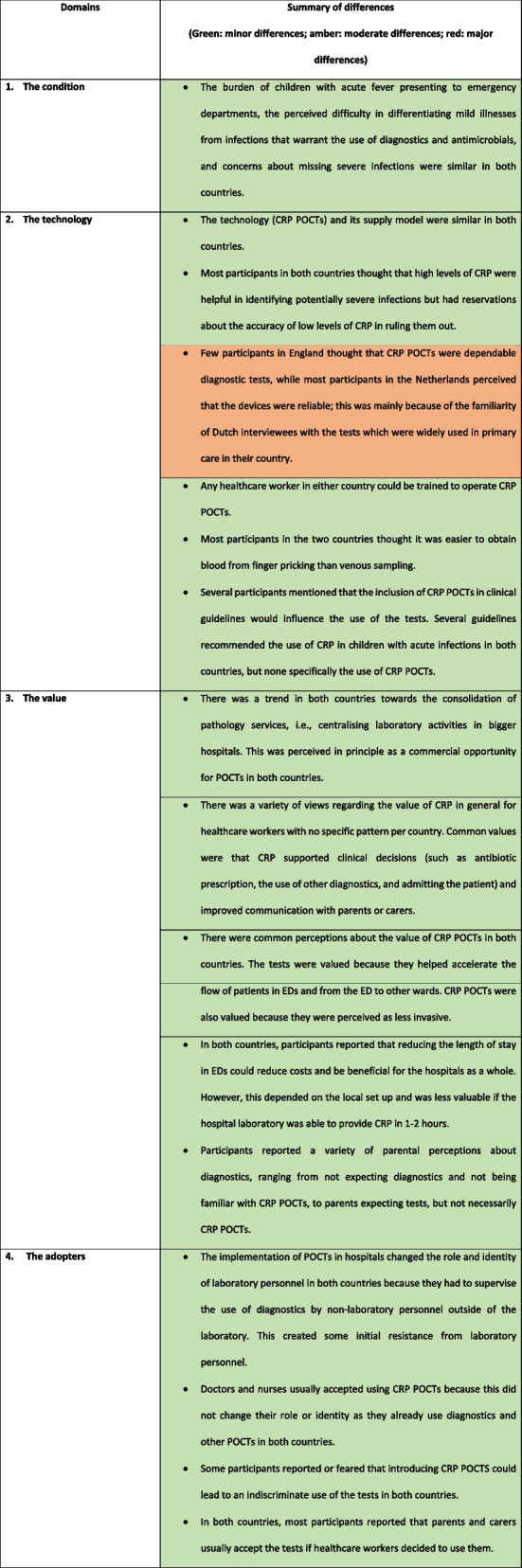

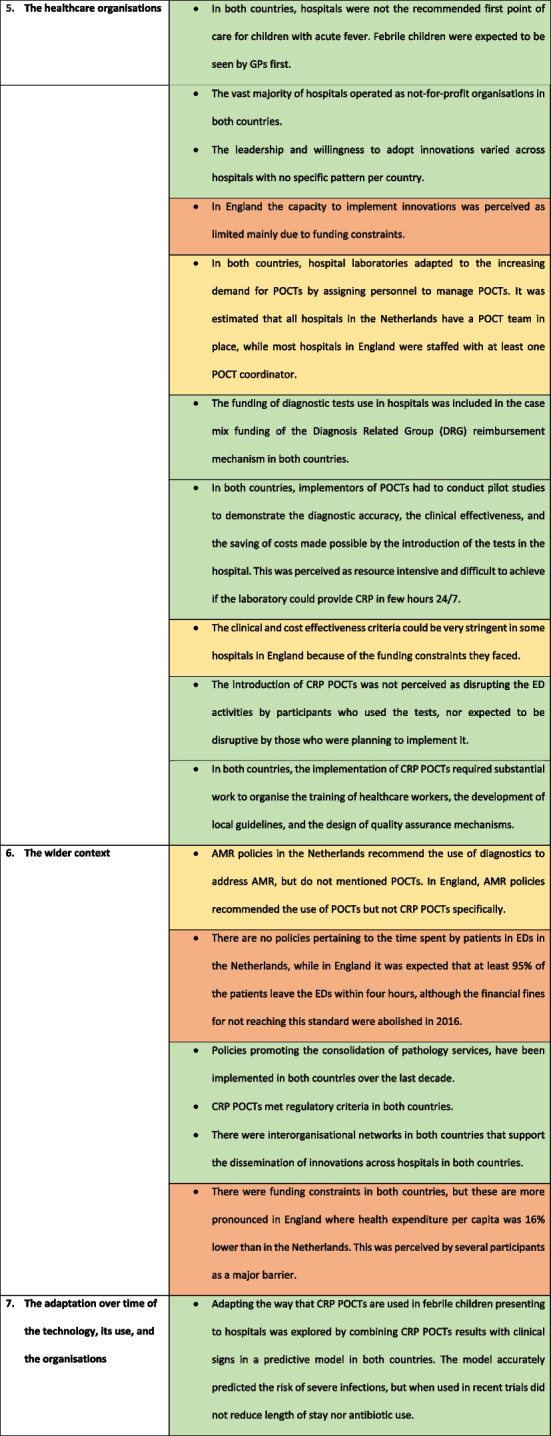
Summary of differences in the NASSS domains that explain the difference in adoption of CRP POCTs in hospitals between the Netherlands and England

*CRP* C-reactive protein, *ED* Emergency Department, *POCT* Point-of-care test, *DRG* Disease related Group

### The condition

The condition of interest was acute fever in children. The review of documents suggested its burden was similar in both countries. Studies estimated that acute fever was the main cause of consultation in hospitals’ EDs, in around 15% of children in the Netherlands [1], and in around 14% in England [2]. Other studies estimated that 0.1–1% of children with acute fever presenting to EDs had severe infections such as septicaemia or meningitis in the Netherlands compared to 1–2.4% in England [20, 21].

Participants in both countries felt that clinically differentiating severe infections from a viral infection is hard, particularly in young infants. Most participants mentioned that because of this, they prescribed antibiotics, used diagnostic tests (“we perform lots of tests that aren’t really necessary”, paediatric infectious diseases doctor-Netherlands), and observed children in hospital for several hours to “cover the bases and to make sure that children are being treated and that nothing (severe) is missed” (nurse 2-England).

### The technology

#### Material features

CRP POCTs were developed in Scandinavian countries [22]. There were 15 different commercially available CRP POCTs. Twelve were quantitative readers and three were semi-quantitative devices [22]. We only considered the quantitative devices because these are the types of devices that have been implemented in the two countries and that were mentioned in the documents included in the documents review. The tests measure CRP levels in whole blood. As only a small volume of blood is required, it can be obtained from a finger prick rather than venepuncture. Additional preparation, such as centrifugation is not required. The drop of blood is place on a cartridge which is plugged into a small mains-powered reader that provides results in around five minutes. In comparison, most participants reported that the turnaround time to obtain results for CRP measured in the hospital laboratory was around one hour in the Netherlands and around two to three hours in England.

A systematic review and meta-analysis found that CRP measured in a laboratory is one of the best biomarkers currently available to identify severe infections in children [23]. However, it can take up to 48 h from the onset of infection before CRP peaks [22]. Because of this delay, most participants in both countries felt that low levels of CRP were not useful to exclude severe infections.

In terms of the accuracy of POCTs devices to measure CRP, several studies showed that the devices were accurate and precise compared to the measurement of CRP in a laboratory [22]. Despite this evidence, few participants in England thought that CRP POCTs were dependable diagnostic tests. By contrast, most participants in the Netherlands perceived that the devices were reliable, and this view was mainly because of the familiarity of Dutch interviewees with the tests: “CRP POCTs are widely used in the General Practice population, so the machines are (already) validated quite properly” (head of emergency department-Netherlands).

#### Types of knowledge generated

Quantitative CRP POCTs provide a measure of blood CRP concentration in mg/L.

#### Knowledge and support to use the tests

Any healthcare professional in the Netherlands and England could be trained to operate the tests. Most participants in both countries thought that using CRP POCTs was easy (“a lot easier in children than trying to get a venous blood sample”, trainee 4-England) and that getting a quick result was a major advantage.

Several participants mentioned that the inclusion of CRP POCTs in clinical guidelines would influence whether they use the tests or not. In both countries, some guidelines for the management of infections recommended using CRP, but not specifically CRP POCTs. Guidelines from the Dutch Royal College of Paediatricians (NVK) recommended the use of CRP in the clinical management of meningitis [24], fever [25], sepsis in children [26], and neonatal sepsis [27]. In England, the National Institute for Health and Care Excellence (NICE) guidelines for meningitis [28], fever in children < 5 years [29], and neonatal infections [29] recommended the use of CRP in similar terms to the Dutch guidelines. The NICE guidelines for urinary tract infection advised against using CRP alone to differentiate between pyelonephritis and cystitis in children [31]. There is also a recent guideline from the Royal College of Paediatrics and Child Health that recommended the use of CRP to decide whether to initiate immunomodulatory therapy in children with COVID-19 [32].

#### Technology supply model

The devices do not need to be locally customized; they are a “plug and play” technology. There were several companies that produced CRP POCTs, several of them being multinational companies that supplied the Netherlands and England [22].

### The value

#### Supply-side value

Some participants reported that there was a trend towards reducing the volume of activities in smaller hospital laboratories and to centralize or consolidate these activities to main hospitals in both countries (see The wider context section). This led to the perception that “there will be more and more point of care in the hospital wards” (in vitro diagnostics industry representative-Netherlands) to cope with this change and suggested that this may increase the commercial value of POCTs in general. In the Netherlands, some participants felt that this trend facilitated the implementation of POCTs. By contrast, in England there was more diversity of views with few participants reporting that consolidation of pathology services promoted the implementation of POCTs, while an industry representative felt that the business case for POCTs has not “stacked up” yet and that even though the diagnostics industry was in principle interested in investing in POCTs, “there needs to be (more) demand” (in vitro diagnostics industry representative-England), which suggested slightly more uncertainty about the commercial value of POCTs.

#### Demand-side value

There were mixed views regarding the value of CRP and CRP POCTs for healthcare workers, with no particular differences between the two countries.

Some participants thought that CRP could help clinical decision making, such as whether or not to prescribe antibiotics, use additional diagnostic tests, and whether to admit or discharge patients, particularly in those with no clear focus of infection. CRP was also perceived by some participants as useful when communicating with parents or carers to reassure them and support decisions.

In terms of CRP POCTs, one participant reported that the tests allowed “decision making a lot quicker” (nurse 3-England), a value that was shared by most participants. Another commonly cited value was that finger pricking was less invasive than venous sampling. The need for only “a few drops of bloods” (paediatric infectious diseases doctor-Netherlands) was also valued by most participants. However, some participants mentioned that this did not apply to complex clinical cases: “(in complex cases) you would normally do the whole shebang (other diagnostics) rather than just do the screening test (CRP POCT)”; Trainee 12 – England). Few paediatricians mentioned that with the use of POCTs, including CRP POCTs, laboratory sampling errors (labelling errors, or loss of samples) might be reduced, although other participants pointed out that these were rare events.

In terms of the value of CRP POCTs at the hospital-level, several participants mentioned that the use of CRP POCTs helped “getting people through quickly” (head of emergency department-Netherlands) in the ED and between the ED and other services. This in turn freed capacity (rooms, beds, availability of healthcare workers) and was particularly important for smaller EDs which struggle to manage high volumes of patients in busy periods of the year. Some participants in both countries also suggested that CRP POCTs could be particularly valuable in smaller hospitals that had scaled back laboratory activities or did not have an onsite laboratory out of hours. In those settings, allowing the ED personnel to use CRP POCTs could be cheaper than having, for example, a laboratory technician on call. To the best of our knowledge, there were no cost-effectiveness evaluations of the use of CRP POCTs in hospitals in children. A cost-saving assessment of a pilot study in England found that using CRP POCTs in children attending the ED resulted in a reduction in the length of stay in EDs and annual savings of more than £60,000 across three hospitals, mainly through reduction of clinicians’ workload [33]. However, the value of accelerating patient flow was thought to be context dependent. Most participants reported that their hospitals were able to provide CRP results from the laboratory in a few hours and some thought that the accuracy of results from the laboratory were of “much higher standards” (head of laboratory 2-Netherlands) than from POCTs. Because of this, several healthcare workers thought that the longer turnaround times for samples analysed in the hospital laboratory compared to the POCTs were acceptable.

In terms of the value of CRP POCTs for parents of febrile children, few participants reported that there was a “massive variety of parental expectations” (trainee 4-England). In both countries, parents were not usually familiar with CRP POCTs. Although parents of children with multiple comorbidities and children referred by a GP tended to expect more diagnostics in general, this does not extend to CRP POCTs.

### The adopters

In this study, the adopters were healthcare workers. Healthcare workers involved in the use of CRP POCTs in children are hospital nurses, paediatricians (including specialist trainees), and laboratory personnel. In both countries, the introduction of POCTs in hospitals changed the role of laboratory personnel, because they had to supervise the use of diagnostics outside of the laboratory and take “full responsibility, including the training, the quality control… everything” (head of laboratory 1-Netherlands). This generated some initial resistance towards POCTs as it increased the workload of laboratory staff.

In England, the implementation of CRP POCTs in a pilot study at one of the hospitals included in this study did not change nurses or doctors’ roles or identity because they already used other POCTs. In the Netherlands, CRP POCTs were about to be introduced in one of the hospitals, and the implementors expected that most staff would accept using the tests because of their routine use of other POCTs. Few participants reported that there might be some resistance from more senior nurses who were less inclined to adopt innovations.

A change in practice feared by some participants in both countries was that introducing CRP POCTs would lead to healthcare workers overusing the tests: “Before you know it, it would get out of hand maybe, and you need to do the test in every patient who comes with a runny nose” (paediatric infectious diseases doctor-Netherlands). This happened in the hospital in England where the tests had been piloted: “it did eventually become used indiscriminately which was a problem” (head of emergency department-England) and was one of the reasons for the test being abandoned after the pilot.

#### Acceptability by patients and carers

None of the participants in either country reported that parents and children refused POCTs, including CRP POCTs. One participant believed that this was because “parents put great faith in technology” (trainee 9-England).

### The organisations

The organisations considered in this study were hospitals. In both countries, parents and other carers of children with acute infections were expected to initially seek medical care at GP practices, as GP are the gatekeepers of health services [34, 35]. However, in both countries some patients did present directly to hospitals [1, 36], usually at the ED. Most hospitals operated as not-for-profit organizations in both countries [37].

#### Capacity to innovate

There were mixed views in terms of the leadership and willingness to adopt innovations. In both countries, few participants reported that this varied across and within workplaces: “it completely depends on the person (in charge)” (head of laboratory 1-Netherlands). In terms of resources, resource constraints were commonly mentioned, but this was particularly the case in England where implementing innovations was perceived as difficult mainly due to funding constraints (see The wider context section).

#### Readiness for the implementation of CRP POCTs

Over the last decade, hospital laboratories in both countries have progressively assigned specific personnel to oversee the use of POCTs to address the increasing demand for POCTs in general. In the Netherlands, a recent cross-country evaluation of quality assurance of POC testing estimated that most hospitals have a POCT team in place [38]; in England, a survey of NHS trusts found that this was the case in 70% of the surveyed hospitals [39]. This may have increased the readiness to implement POCTs, although one participant in England reported that many hospitals actually have only one person in charge of POCTs (rather than a team) and suggested that this person was sometimes overwhelmed, which might be a barrier to the implementation of POCTs.

#### Funding decision

The funding of diagnostic tests in hospitals was included in the case mix funding of the Diagnosis Related Group (DRG) reimbursement mechanism in both countries, called Diagnosis Treatment Combination system (DOT-DBC) in the Netherlands and Payment by Result in England [34, 35]. In both countries, clinical cases were classified into groups which comprise cases that were clinically similar and were homogenous in terms of resource use (e.g., medical and surgical procedures, severity, length of stay). The sum of money that was reimbursed for providing care to each group, including the use of diagnostics, was set in advance by the Dutch Health Authority (NZA) in the Netherlands and by the Department of Health in England [40, 41], based on average costs of care for each clinical condition across all hospitals. Each group was assigned a code and hospitals billed the codes generated through their activity to the funder of hospital care. In the Netherlands funders were not-for-profit health insurance companies, while in England they were clinical commissioning groups (replaced by Integrated Care Boards from July 2022), which were public organisations funding primary and hospital care for the population of a geographical area. Under this system, hospitals received a fixed sum of money per case, regardless of the number of diagnostic tests used. This incentivised hospitals to limit their expenses for each case to ensure they do not exceed the reimbursement they receive. This may have discouraged the use of CRP POCTs which are more expensive than CRP measured in the laboratory, except if using the tests reduced costs elsewhere by, for example, reducing length of stay. In both countries it was necessary to present a business case with the potential cost savings generated by introducing the tests in the hospital care pathways “to justify the costs of CRP POCTs” (general paediatrician 2-England). Moreover, pilot studies were required to demonstrate the diagnostic accuracy of POCTs compared to the laboratory equivalent. In England, some participants reported that the level of evidence needed to justify the adoption of new diagnostics varied across hospitals and was sometimes very stringent. A recent workshop by the Academy of Medical Science to explore the future of diagnostics in the NHS reported that barriers to the adoption of diagnostics included hospitals requirement for the same level of evidence for diagnostics as for pharmaceuticals, while the clinical trial research infrastructure was less developed for diagnostics than for pharmaceuticals [42].

#### Disruption in team routines and interactions

Using POCTs in general was not seen as disruptive in both countries, even if patient care “ takes a bit more time” when POCTs are used (nurse 2-England).

#### Work needed to implement change

Several participants in both countries mentioned that the work needed to implement the tests after hospital-level approval was substantial and often underestimated: “it sounds simple but the administration, the quality you have to ensure, the maintenance… that’s very demanding. People underestimate the time you need for all of this” (head of laboratory 3-Netherlands).

### The wider context

#### Policy context

Policies pertaining to antimicrobial resistance (AMR) were examined because an expected impact of CRP POCTs is the reduction of antibiotic use. In hospitals, alternatives to CRP POCTs to reduce antibiotic prescription, such as laboratory-measured CRP, microbiology, and observing/admitting the patient were available; however, in busy periods of the year, CRP POCTs may have helped to expedite the decision to prescribe antibiotics or not. The Dutch AMR policies recommended the use of new diagnostics in general to mitigate AMR but does not specifically mention POCTs [43]. In England, the UK AMR policy supported the use of POCTs, but did not mention CRP nor any specific biomarkers [44].

Policies pertaining to the time spent by patients in EDs were also examined because several participants mentioned that improving the flow of patients was one of the most important potential values of CRP POCTs. In the Netherlands, there was no such policy [45]. In contrast, in England, the NHS has introduced waiting time standards in 2004 to reduce ED overcrowding. Their aim was that 95% of people attending ED were seen within four hours [46]. Hospitals that did not reach those targets endured a financial fine. One head of an ED in England mentioned that this was an important reason to pilot the test in his department. The fines were removed in 2016, but the 4-h limit remains as a standard for English ED services [47].

We also examined strategies for consolidation of laboratory services, as some participants reported that laboratory consolidation was a driver of POCTs implementation. There were no substantial differences between the two countries. In England, following the publication of two independent reviews [48] the NHS promoted the centralisation of some laboratory analyses in central hubs to reduce the cost of pathology services [49]. Similarly, this approach was also adopted in other European countries during the last decade, including the Netherlands [50, 51].

#### Economic context

Containment of healthcare costs is a common challenge across European countries, particularly since the 2008 economic crisis [52]. However, cost-containment has been particularly important in the UK [34, 52]. As a result, health expenditure per capita in the UK was 16% lower than in the Netherlands [53], and several participants reported that containment of healthcare cost was an important barrier to the introduction of innovations in general in the NHS.

#### Regulatory context

The 12 quantitative CRP POCTs were CE marked in accordance with the European Union IVD Directive (98/79/EC) [22]. CE marking is a process through which the manufacturer self-declares that the device conforms with EU regulatory standards [54]. This allowed manufacturers to commercialise the tests legally in the EU, including the Netherlands and England (until December 2020 for the latter).

#### Role of professional bodies

As mentioned earlier, the use of CRP was recommended in guidelines from the Dutch Paediatric Association, NICE, and the RCPCH, although none mention the use of CRP POCTs specifically. The role of these bodies in both countries on hospital adoption of tests such as CRP POCTs was limited because the inclusion of a relatively cheap diagnostic test (cheap compared to, for example, the use of CT-scan) in a guideline had limited influence on the definition of the DRG reimbursement groups and their price [41].

#### Interorganisational networks

In both countries, few participants mentioned that they exchanged knowledge and experiences about the introduction of new diagnostics through informal and formal professional networks. Among the formal organizations, there were regional support structures that help disseminate healthcare innovations, such as ROS Robuust in the Netherlands and the Academic Health Sciences Network in England [55, 56]. The Oxford AHSN led the pilot study in three English hospitals mentioned in The value section.

### Adaptation over time of the innovation, its use and the organisations

CRP POCTs devices could not be physically changed or adapted. However, there have been attempts to modify the use of CRP POCTs by incorporating the tests into a clinical tool that predicted the risk of severe infections in febrile children presenting to EDs, combining clinical signs and CRP results in one score. One such study including Dutch and English febrile children, accurately predicted the risk of severe infection [20]. However, the use of the tool did not reduce length of stay or antibiotic use in febrile children in two recent trials conducted in the Netherlands [57, 58].

## Discussion

### Summary of principal findings

Our study suggests that the main explanators of the higher availability of CRP POCTs in hospitals in the Netherlands compared to England lie at the micro and macro levels of health systems. Most hospital healthcare workers in the Netherlands are familiar with CRP POCTs because the tests are widely used in primary care, and healthcare workers often see patients referred by GPs with CRP POCTs results. This familiarity made most hospital healthcare workers believe that CRP POCTs are dependable diagnostics. In contrast, in England, where the tests are less available in primary care, most participants expressed doubts about the reliability of the technology. This is an important difference because healthcare workers usually initiate the process of implementing new diagnostics.

In terms of the macro level, although hospital diagnostics are funded through similar Diagnosis Related Group reimbursement mechanisms in the two countries, the actual funding for healthcare is more constrained in England. This can result in more scrutiny and the use of stricter clinical and cost-saving criteria during the decision-making process to adopt diagnostic tests. This can in turn lead to the multiplication of pilot studies and is an important barrier to the implementation of new diagnostics, including CRP POCTs.

There are neither substantial nor consistent differences between countries in terms of the burden of the condition, the value of CRP POCTs for industry, users or patients, and the impact of CRP POCTs on the identity or practices of healthcare workers. Hospitals adapted to the increased demand for POCTs in both countries by assigning laboratory personnel to manage POCTs outside of the laboratories, although this process seems more advanced in the Netherlands. There are similarities and differences in terms of high-level policies and standards. The consolidation of laboratory services has been promoted in the two countries over the last decade in a similar way. However, the AMR policies differ: in England policies recommend the use of POCTs (although not specifically CRP POCTs) while in the Netherlands they only mention diagnostics in general. There are standards regarding the time spent in EDs in England, but there is no equivalent in the Netherlands. The AMR policy and ED attendance time standards could have led to more adoption of CRP POCTs in England than in the Netherlands; the fact that this did not happen suggests that there may be a disconnect between high-level policies and what effectively happens in health services, and/or that the introduction of new diagnostic tests is comparatively more difficult in England.

Although we primarily examined the reasons for the different levels of adoption of CRP POCTs in hospitals in the Netherlands and England, it is worth noting that the tests are less often adopted in hospitals than in primary care in both countries. Our study suggests that this is because in most hospitals, laboratory-measured CRP provides an alternative to CRP POCTs. In addition, hospitals receive a fixed sum of money for each clinical case via the Diagnosis Related Group funding mechanism. This encourages hospitals in both countries to use fewer and cheaper diagnostics to ensure the reimbursement covers the actual cost of care, which favours laboratory CRP, as it is cheaper than CRP POCTs. However, CRP POCTs can be useful in other hospitals, such as hospitals where the laboratory cannot provide CRP levels 24/7, hospitals where the turnaround time is long, which affects the flow of patients in EDs, and hospitals where the ED resources (personnel and infrastructure) are limited and expediting patient care is particularly important. The higher availability of CRP POCTs in hospitals in the Netherlands compared to England presumably takes place in those types of hospitals.

### Comparison with other literature

In the Netherlands, a survey of GPs found that 80% of GPs use CRP POCTs [59], and it has been described that there is a strong integration between primary and secondary care with most hospitals involved in the provision of services to primary care [60], including the implementation of CRP POCTs in GP practices. The widespread adoption of CRP POCTs in primary care and the better integration of primary and secondary care supports our finding that hospital healthcare workers in the Netherlands are more familiar with CRP POCTs than in England.

This study suggests that introducing POCTs was more challenging in England than in the Netherlands. In line with this finding, the most recent UK National Action Plan against AMR indicates that the adoption of diagnostics in the NHS was difficult and that “if a new promising diagnostic came out tomorrow, the NHS is not equipped to get it into front-line use quickly” [61]. Funding constraints in England were identified as an important barrier to the implementation of CRP POCTs in this study. An independent review of the introduction of innovations in the English NHS found that funding restrictions were limiting the adoption of innovations. That review found that hospitals need to prioritise investment in innovations, which leads some hospitals to apply high standards of clinical and cost-effectiveness, “sometimes hardly attainable”, before deciding to adopt an innovation [62], which is in keeping with our results. Another report describing child healthcare in the UK suggests that this may even result in some rationing of care [63]. A recent qualitative study about the barriers to the implementation of POCTs in England found that cost was one of the two most cited barriers [64].

As mentioned earlier, we conducted a previous qualitative study to understand the factors that contribute to a greater availability of CRP POCTs in the Netherlands in primary care [12]. The main factors lied also at the micro and macro levels of health systems, but were different. At the micro level, the generation of robust evidence about the effectiveness of the tests combined with strong advocacy efforts of early adopters played a key role. At the macro level, the role of clinical guidelines and their developers in determining which interventions are re-imbursed in primary care and the operational support from laboratories to GP practices were decisive factors that led to the greater adoption of the tests in primary care the Netherlands.

### Strengths and limitations

To the best of our knowledge, this is the first study to comprehensively compare the adoption of CRP POCTs in hospitals in two countries. Using the NASSS framework allowed us to conduct an in-depth, wide-ranging, and consistent comparative health systems analysis. We conducted a document analysis in combination with interviews of a wide range of stakeholders in the two countries which allowed us to triangulate the findings presented in this article. Moreover, most studies on the adoption of CRP POCTs focus on the adoption of tests in adult patients in primary care; this is one of few studies focusing on the adoption of tests for the management of acute childhood infections in hospitals. Our findings should be interpreted in light of some limitations. We were unable to interview children and their carers, whose contributions could have provided important additional information. Moreover, the backgrounds and experience of using POCTs by some of the authors may have influenced the interpretation of data towards a positive perception of the role of diagnostics and POCTs in clinical practice, despite the best attempts to limit this.

### Implications for organisations implementing POCTs and future research

Organizations considering implementing POCTs in hospitals should carefully consider how the implementation of the tests realistically fits with the potential users’ perceptions of dependability and utility, and with the reimbursement mechanisms for diagnostics. However, the resources needed to do this can be substantial and are not available to all stakeholders, particularly those working at the micro level, such as frontline paediatricians. Large multidisciplinary research consortia or large diagnostic test companies may have more resources to undertake such a big task. Collaboration between as many relevant stakeholders as possible is needed to comprehensively assess the relevant factors in a given country.

The cost-effectiveness of CRP POCTs compared with traditional central laboratory testing in the management of acute childhood infections in the ED is unclear and warrants further evaluation and should incorporate a range of outcomes both at the level of the individual patient and health services. Additional comparative analyses with other POCTs in other countries with different health systems arrangements would be useful to provide further insights to inform the implementation of current and future POCTs.

## Conclusion

CRP POCTs appear to be more widely available in hospitals in the Netherlands because of the greater familiarity of Dutch healthcare workers with CRP POCTs and because there are more funding constraints in England. Most hospitals in the Netherlands and England have not adopted CRP POCTs because the alternative CRP measurements from the hospital laboratory are available in a few hours and at a lower cost.

### Supplementary Information

**Additional file 1. **Topic Guide (doctors): Adoption of C-reactive protein rapid tests in hospital.

## Data Availability

The datasets generated and/or analysed during the current study are available in the London School of Hygiene and Tropical Medicine Compass data repository (https://datacompass.lshtm.ac.uk).
