# Supplementary material for: Adoption of C-reactive protein rapid tests for the management of acute childhood infections in hospitals in the Netherlands and England: a comparative health systems analysis
Source: BMC Health Serv Res. 2024 Mar 19;24:351. doi: 10.1186/s12913-024-10698-6 (PMC10949747; doi:10.1186/s12913-024-10698-6)
Supplement: Supplementary file 1 — Additional file 1. Topic Guide (doctors): Adoption of C-reactive protein rapid tests in hospital. [file 12913_2024_10698_MOESM1_ESM.pdf]

Topic Guide (doctors): Adoption of C-reactive protein rapid tests in hospitals

Participant ID Number:	Gender: Male / Female
Country:	Date (DD/MM/YY):

Introduction:

- Overview and purpose of study
- Who is involved
- Aims of interview and expected duration
- Why participant has been selected
- What will happen to the results of this study
- Questions
- Go through consent form with participant

Warm-up and general information:

What is your role in the hospital?

In which department(s) do you work?

When did you graduated?

Which rapid POCTs are available? How many devices are available?

Turnaround time for routine laboratory tests (eg CRP, full blood count, chemistry)?

<table>
<tr><th>Topics</th><th>Questions</th></tr>
<tr><td>1. Current practice</td><td>A 4-month-old infant presents with fever, asymptomatic otherwise, clinical examination unremarkable. The infant was inconsolable all morning, but currently settles with mum and feeding well, 3 non-bilious, milky vomits, vitals are within normal limits. Mum says he never vomits after feeds.

• How would you manage this patient?

• What are challenges, if any when seeing a child with acute fever?

• What factors influence your decision to:
  o Discharge the patient home or admit into the hospital?
  o Use/not use antibiotics?
  o Use diagnostic tests? If yes, which tests? And why?</td></tr>
<tr><td>2. The technology and its value</td><td>• Have you used CRP POCT?

  • If yes in which circumstances?

  • What were the advantages/disadvantages of using CRP POCT?
    ▪ For you?
    ▪ For the department(s) where your work?

  • Have you used them in children?
    ▪ If yes, what were the advantages/disadvantages of using CRP POCT in children?
    ▪ If no, why?

  • How did parents/children perceive the use of CRP POCT?

• If no, why?

• If because tests are not available, let's imagine the test are made available.

  • In which circumstances would you use the tests?

  • What would be the advantages/disadvantages of using CRP POCT?
    ▪ For you?
    ▪ For the department(s) where your work?

  • Would you use them in children?
    ▪ What would be the advantages/disadvantages of using CRP POCT in children?
  • How would parents/children perceive the use of CRP POCT?</td></tr>
</table>

3. The adopters and the impact of CRP POCTs	• What changes, if any, did the use of CRP POCT brought/would bring to:<br>○ the way you work?<br>○ Your role in the department/hospital?<br>• Was the test accepted/ would the test be accepted by parents/children?<br>• Why yes/no?
4. The department/hospital	• How innovative in general is the department where you work? The hospital?<br>• Can you tell me about an innovation that was introduced when you were working here? What happened?<br>• How ready was/is the department/hospital for the introduction of CRP POCT?<br>• What problems did you encounter/would you encounter in the implementation of CRP POCT?<br>• Who decided/would decide whether the test should be adopted?<br>• What are the criteria to decide to adopt tests such as CRP POCT?<br>• How are/would be the cost of using the test be covered?<br>• What impact did/would the use of CRP POCTs have on the way your department is organised? On the relation with other departments (eg the lab)?<br>• What work was/would be needed to adopt the test once the decision to implement it is taken?<br>• What were/could be the main challenges in this process?
5. The wider context	• Are you aware of the AMR policy of your country?<br>• What impact does it have on your willingness to implement/use CRP POCT?<br>• What impact does it have on your prescription of antibiotics?<br>• Are there other policies that have an impact on the use of diagnostics/antibiotics?<br>• What about the 4-hour waiting time policy?<br>• What role, if any, did your professional association had /could have on the process of implementing tests such as CRP POCT?<br>• How do you get to know about innovations? How is the knowledge about innovations disseminated across departments/hospitals?

6. Adaptation over time	• Has the use of CRP POCT changed since you started using it? Why?<br><br>• How do you think the use of the tests would evolve if you started using it?<br><br>• What could change the availability and use of CRP POCTs in the future?

- Ask participant if he/she has any question
- Ask if there is another relevant person he/she would recommend interviewing
- Ask if there is any document/website he/she would recommend accessing
- Thank participant.
